# *Opuntia* cladode powders inhibit adipogenesis in 3 T3-F442A adipocytes and a high-fat-diet rat model by modifying metabolic parameters and favouring faecal fat excretion

**DOI:** 10.1186/s12906-020-2824-x

**Published:** 2020-02-05

**Authors:** Cécile Héliès-Toussaint, Edwin Fouché, Nathalie Naud, Florence Blas-Y-Estrada, Maria del Socorro Santos-Diaz, Anne Nègre-Salvayre, Ana Paulina Barba de la Rosa, Françoise Guéraud

**Affiliations:** 1 INRA, ToxAlim (Research Centre in Food Toxicology), Université de Toulouse, INRA, ENVT, INP-Purpan, UPS, Toulouse, France; 2 Centro de Investigación y Estudios de Posgrado (CienciasQuímicas), UniversidadAutónoma de San Luis Potosí, San Luis Potosí, Mexico; 3 Instituto Potosino de Investigación Científica y Tecnológica, San Luis Potosí, Mexico; 4 INSERM U1048, Institute of Metabolic and Cardiovascular Diseases I2MC, Toulouse, France

**Keywords:** *Opuntia*, Anti-obesity, 3T3-F442A adipocytes, Rat high-fat diet, Faecal fat excretion

## Abstract

**Background:**

Obesity is a major public health concern worldwide. A sedentary life and a nutritional transition to processed foods and high-calorie diets are contributing factors to obesity. The demand for nutraceutical foods, such as herbal weight-loss products, which offer the potential to counteract obesity, has consequently increased. We hypothesised that *Opuntia* cladodes consumption could assist weight management in an obesity prevention context.

**Methods:**

This study was designed to explore the anti-adipogenic effects of lyophilised *Opuntia* cladode powders (OCP) in an in vitro cellular model for adipocyte differentiation and an in vivo high-fat-diet (HFD)-induced obesity rat model. Two OCP were tested, one from wild species *O. streptacantha* and the second from the most known species *O. ficus-indica*.

**Results:**

Pre-adipocytes 3 T3-F442A were treated by OCP during the differentiation process by insulin. OCP treatment impaired the differentiation in adipocytes, as supported by the decreased triglyceride content and a low glucose uptake, which remained comparable to that observed in undifferentiated controls, suggesting that an anti-adipogenic effect was exerted by OCP. Sprague–Dawley rats were fed with a normal or HFD, supplemented or not with OCP for 8 weeks. OCP treatment slightly reduced body weight gain, liver and abdominal fat weights, improved some obesity-related metabolic parameters and increased triglyceride excretion in the faeces. Taken together, these results showed that OCP might contribute to reduce adipogenesis and fat storage in a HFD context, notably by promoting the faecal excretion of fats.

**Conclusions:**

*Opuntia* cladodes may be used as a dietary supplement or potential therapeutic agent in diet-based therapies for weight management to prevent obesity.

**Graphical abstract:**

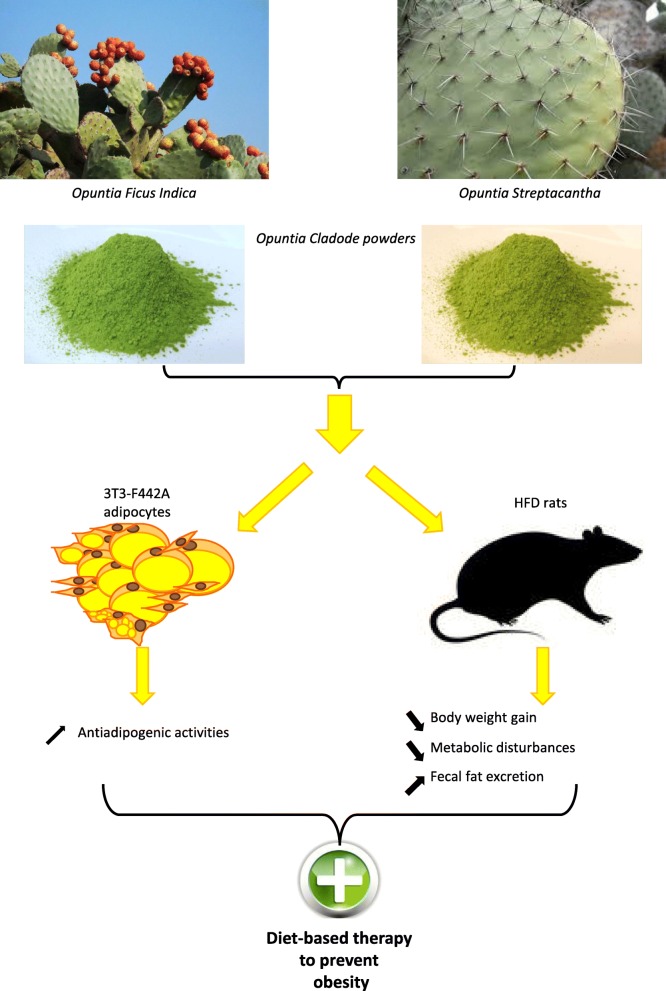

## Background

Obesity is a serious health issue of global concern that not only decreases life span but is also associated with the development of many chronic illnesses, such as cardiovascular diseases, type 2 diabetes, hypertension, fatty liver disease and cancer. It is considered the fifth risk of death worldwide [1, 2].

In 2017, Mexico had the highest global prevalence of obesity in the adult population (15–74 years, 32.4%), while countries like Japan or Korea had the lowest incidence (below 6%). Accelerated urbanisation, the improvement in socioeconomic conditions, and dietary changes are considered responsible for the dramatic and rapid increase in obesity prevalence. Traditional food consumption has decreased in favour of increased consumption of refined foods, as well as sweets and fat/sugar-rich diets [3]. With obesity rates projected to increase further by 2030, new strategies and cost-effective actions are needed for the prevention and control of obesity in children, adolescents and adults [4]. Promoting healthy lifestyles in the population is a major challenge for the health sector. Comprehensive public policies aimed at reversing the trend worldwide are required, particularly in Mexico.

Current anti-obesity agents often present disadvantages, while disappointing results may be observed after the arrest of lifestyle modification or pharmacotherapy, indicating a need for alternative treatment modalities that produce better and long-term results of obesity prevention or weight management [5]. Thus, identifying efficient and easy-to-use agents is a priority for medical research. Herbal supplements and diet-based therapies for weight loss are among the most common, complementary and alternative therapeutic modalities [6–8]. In Mexico, numerous plants have been identified and used in folk medicine to prevent and cure chronic diseases. Among them, *Opuntia* spp., including the fruit, stems, seeds and cladodes, exhibit diverse health benefits and high biotechnological potential. *Opuntia* cladodes are a good source of dietary fibers, which contributes to reducing body weight [5, 9–11], and the presence of antioxidants could be responsible for the nutritional and protective benefits of *Opuntia*-enriched diets in chronic diseases [6]. *Opuntia* is a species of cactus native to Mexico. Besides its medicinal purposes, it has been domesticated or naturally selected for food and ornamental use [6]. Scarce information is available concerning the effects of domestication on the biological properties of *Opuntia*, at the molecular and biochemical levels. In previous studies, we highlighted the variations in chemical composition and the anti-atherogenic and anti-tumoral properties of various wild and domesticated *Opuntia* varieties [12–14], including *O. streptacantha* (OSC),the wildest variety, followed by *O. hyptiacantha*, *O. megacantha*, *O. albicarpa*and *O. ficus-indica* (OFI), which is the most known and cultivated species, with the highest degree of domestication.

Rodent studies have shown that *Opuntia* extracts modify obesity biomarkers. In Zucker obese rats, Nopal consumption attenuated hepatic steatosis related to obesity and reduced obesity-related metabolic abnormalities. Vinegar or isolated molecules present in *Opuntia* cladodes, such as kaempferol or isorhamnetin, used in obese mice models, corroborated the anti-obesity and anti-diabetic potentials of these molecules [15–21]. These studies suggest that remarkable effects could be observed in rat/mouse models of obesity induced by a high-fat diet (HFD), supplemented with *Opuntia* cladode powders (OCP). Further results with animal models are required to understand the underlying molecular mechanisms for these effects.

Adipose tissue growth occurs because of an increase in the size of existing adipocytes or the number of adipocytes. An imbalance between energy intake and energy expenditure generates an excess in adipose tissue resulting in obesity. Differentiation of pre-adipocytes into adipocytes involves a comprehensive network involving transcription factors responsible for the expression of key proteins that induce mature adipocyte formation. Adipogenesis also involves changes in cell morphology, induction of insulin sensitivity, and changes in β-cell secretory capacity. Deciphering the mechanism of how certain nutrients affect adipocyte differentiation and adipogenesis is important for the prevention of obesity and related diseases [22].

The present study was designed to investigate whether *Opuntia* species exert anti-obesity properties by examining the anti-adipogenic effect of two *Opuntia* cladode powders (OCP) and elucidating the mechanisms underlying such effects. For this purpose, we used powders from the wildest (OSC) and the most domesticated (OFI) *Opuntia* varieties. We chose these two species for their highest domestication gradient difference (from the ancestor specie (OSC, growing in wild habitat) to the most propagated *Opuntia* for commercial production (OFI). The differentiation of pre-adipocytes into adipocytes was examined by treating 3 T3-F442A cells [22] with OCP to investigate the effects at the cellular level. The same powders were also tested on an animal model of obesity by feeding Sprague–Dawley rats with a high-fat diet (HFD) supplemented or not with the powders. This animal model allowed us to examine the whole-body level, as the first step towards human trials.

## Methods

### Reagents

Cytochalasin B, 2-deoxyglucose, insulin and Dulbecco’s modified Eagle’s medium (DMEM) were all purchased from Sigma–Aldrich (Saint-Quentin-Fallavier, France). [^3^H]-2-deoxyglucose was from PerkinElmer (Boston; WalthamMA, USA).

### Opuntia plant material and cladode powder preparation

*Opuntia* young cladodes from the wild species OSC (*O. streptacantha* Lem., cv. Tuna Loca) and OFI (*O. ficus-indica* [L.] Mill., cv. RojoVigor) were collected (April 2010 and 2012) from the *Opuntia* Germplasm Bank of the Agrobotanical Garden located in El Orito, Zacatecas, Mexico. The formal identification of the plants was reported by Ramirez-Tobias et al. [23]. The plants were grown under the same environmental conditions [12]. Cladodes were washed, ground in liquid nitrogen using a KrupsGX 4100 grinder (Mexico City, Mexico), and kept at − 80 °C until further processing. Samples were freeze-dried (Labconco, Kansas City, MO, USA), sieved through mesh 80, then stored in plastic bags at 4 °C until use [14]. Contents of fat, crude fiber and total phenolic compounds were determined, as previously reported [12].

### Cell culture and adipocyte differentiation

#### Cell culture

The 3 T3-F442A cell line was a gift from Prof. P. Valet (I2MC, Toulouse, France). Pre-adipocytes were cultured in DMEM with penicillin (100 UI/mL)–streptomycin (0.1 mg/mL) (Sigma–Aldrich, Saint Quentin Fallavier; France) supplemented with 10% foetal calf serum (Gold Serum, PAA Laboratories, Les Mureaux, France). Cells were cultured at 37 °C in a 5% CO_2_ humidified atmosphere. Differentiation was induced by incubating confluent 3 T3-F442A cells in a differentiation medium (DMEM supplemented with 10% foetal calf serum and 50 nM insulin) for up to 10 days, with the culture medium renewed every 2–3 days. The non-insulin-treated cells were considered as non-differentiated controls. Treatments with *Opuntia* powders, prepared as described above, were applied during the 10 days of differentiation, concomitantly with insulin treatmentFor all in vitro experiments, the two OCP were diluted directly in the culture medium (100 μg/mL), mixed vigorously, and filtered at 0.2 μM before dilution in DMEM to 1, 10 and 100 μg/mL. The culture medium was changed every 48 h, renewing OCP at the corresponding concentrations in the medium. Cells were seeded at 7.5 × 10^4^ cells/well in 6-well plates for triglyceride (TG) content evaluation, at 2 × 10^4^ cells/well in 24-well plates for glucose uptake assays and at 5 × 10^3^ cells/well in 96-well plates for cytotoxicity (3-[4,5-dimethylthiazol-2-yl]-2,5-diphenyltetrazolium bromide, MTT).

##### Triglyceride (TG) assay

The intracellular TG was quantified using the TG PAP 150 enzymatic kit (TG PAP 150, BioMérieux, Marcy l’Etoile, France) after cell lysis (0.1 N NaOH). To account for cellular proliferation or toxicity of the molecules, the TG content was related to the protein content, determined by the bicinchoninic acid (BCA) enzymatic kit (Pierce, Thermofischer Scientific, Bordeaux, France). The results were expressed as the percentage of control cells treated only with insulin (50 nM).

##### Glucose uptake assay

Glucose uptake was measured according to Kim et al. [24] with some modifications [25]. Briefly, after 10 days of treatment, cells were washed twice in serum-free DMEM and pre-incubated in this medium at 37 °C for 16 h. After this starvation period, cells were washed twice with Krebs–Ringer bicarbonate buffer (KRB) and incubated at 37 °C for 30 min with 100 nM insulin (or not, for the negative control). To initiate glucose uptake, 2-deoxy-[1-^3^H]-glucose (1 μCi/mL) diluted in 0.1 mMD-glucose solution was added to each well and the plates then incubated at 37 °C for 10 min. After incubation, the cells were washed twice with ice-cold KRB buffer and lysed in 0.1 N NaOH. Half of the content of each well was transferred to scintillation vials, and 10 mL of scintillation cocktail (Ultima Gold, Perkin Elmer, Boston, WalthamMA, USA) was added. The radioactivity incorporated into the cells was measured using a liquid scintillation counter (Hewlett Packard, USA). The BCA protein content was assayed for each point on the remaining half.

##### Determination of cellular toxicity

After incubation, the wells were gently rinsed with cold phosphate-buffered saline, and then 20 μL of 5 mg/mL MTT was added to each well and incubated for 4 h. Subsequently, the media from each well was gently aspirated, and 100 μL of dimethylsulphoxide was added to dissolve the formazan crystals. Plates were shaken for 30 min, followed by absorbance measurements at 570 nm using a Tecan microplate reader (Tecan, Raleigh, USA).

### Animal experiments

#### Animals and diets

Male Sprague–Dawley rats (6-weeks-old, body weight of 200 g) were purchased from Charles River Laboratory (Saint-Germain-Nuelles, France). Rats were housed in polycarbonate cages maintained at 24 °C, with 40–70% humidity and 12/12- h light/dark cycles, with free access to food and water. The rats were housed in groups of two to avoid single animals. The protocol was approved by the local ethics committee (TOXCOM/0011/FG FG). A total of 40 rats were randomly distributed into four groups of 10 animals for the following treatments: control group fed with the standard diet (SD); HFD-fed group (F), and HFD-fed group supplemented with 0.5% w/w OCP OSC (F-OSC) and OFI (F-OFI), respectively. Cages were randomly assigned to the racks, to avoid artefacts related to the position. The experimental diets were fed for 60 days. All diets were based on a modified standard AIN76 diet, prepared and formulated in a powdered form by the Experimental Feeds Preparation Unit (UPAE, INRA, Jouy-en-Josas, France) and stored at − 20 °C. The ingredients and macronutrients composition of the diets (g/kg) are listed in Table 1. The HFD was enriched with 25% lard (Cooper l, Lamballe, France). The lipid percentages are listed in Table 2. The diets were renewed every 2–3 days and distributed randomly. The diet intake and the animals’ body weight were monitored every 2–3 days in the morning, and animal welfare was checked simultaneously. The number of animals per group (*n =* 10) was calculated to obtain statistically significant results for diet-induced changes. The days before the end of the experiment, 24-h faeces were collected and stored at − 80 °C. At the end of the study, the rats were killed by CO_2_ asphyxiation according to the protocol of the French “National Charter Concerning the Ethics of Animal Experimentation”. Briefly, each rat was placed in a 25-L polycarbonate chamber. Then, CO_2_was emitted into the chamber at a flow rate of about 5.5–7.5 L/min until the rat was unconscious. The CO_2_ flow continued for at least 60 s to ensure that the breath was not seen before removing the rat from the chamber. Blood samples were collected from the inferior vena cava for biochemical analysis. Liver and abdominal fat were immediately removed, weighed and stored at − 80 °C.

**Table 1:** Ingredients and macronutrients composition of the diets (g/kg)

Ingredient	SD	F	F-OFI or F-OSC
Casein	400	200	199
Corn starch	150	50	49,7
Sucrose	298	348	346,3
Cellulose	50	50	49,7
Methionin	3	3	3
Minerals	34,8	34,8	34,6
Vitamin mix (type AIN76)	10	10	9,9
Calcium phosphate	2,1	2,1	2,1
Choline bitartrate	2	2	2
Ferric citrate	0.144	0.144	0.14
Safflower oil	50	50	49.7
Lard	0	250	248.7
OCP	0	0	5

SD = standard diet; F = high fat diet; F-OFI or F-OSC= Cladode powder from *O. ficus-indica* (OFI) or *O. streptacantha* (OSC); OCP = *Opuntia* cladode powder.

**Table 2:** Lipid composition in diets

Fat	Saturated	Unsaturated	Other
Safflower oil	10% 7% Palmitic acid (16:0) 3% Stearic acid (18:0)	89% 75% Linoleic acid (18:2 n-6) 14% Oleic acid (18:1 n-9)	1%
Lard (hog fat)	40% 27% Palmitic acid (16:0) 11% Stearic acid (18:0) 2% Myristic acid (14:0)	59% 44% Oleic acid (18:1 n-9) 11% linoleic acid (18:2 n-6) 4% palmitoleic acid (16:1 n-7)	1%

##### Biochemical analysis

Plasma samples were separated from blood cells by centrifugation at 1000×*g* for 15 min. Plasma levels of leptin, insulin and monocyte chemoattractant protein-1 (MCP-1) were analysed by the Luminex kit (RMHMAG-84 K-05) (Thermofisher, Bordeaux, France). TG, glucose and hepatic parameters (aspartate aminotransferase, AST; alanine aminotransferase, ALT) were all measured at the Anexplo Facilities, Toulouse (France). Adiponectin was evaluated using the TECO medical Mediagnost (E091-Rkit, Reutlingen, Germany), and C-reactive protein (CRP) was measured using the Abcam kit (ab108827, Abcam, Cambrigde, UK).

##### Measurement of hepatic and faecal triglyceride (TG) levels

Hepatic lipids were extracted by homogenisation of liver tissues in phosphate-buffered saline. Ethanol was added to the homogenates (1:9, v/v), and the samples were mixed at room temperature for 1 h to solubilise the TG. After centrifugation at 2000×*g* for 10 min, the supernatant was collected to measure the TG level using the TG PAP 150 enzymatic kit (TG PAP 150, BioMérieux, Marcy l’Etoile, France), according to the manufacturer’s protocol. Faecal TG lipids were extracted from the faeces collected over 24 h. The samples were homogenised in sterilised water, then centrifuged at 2000×*g* for 10 min. The supernatant was collected to measure the TG level using the TG PAP 150 enzymatic kit (TG PAP 150, BioMérieux, Marcy l’Etoile, France), according to the manufacturer’s protocol.

### Statistical analysis

**Fig. 1:**
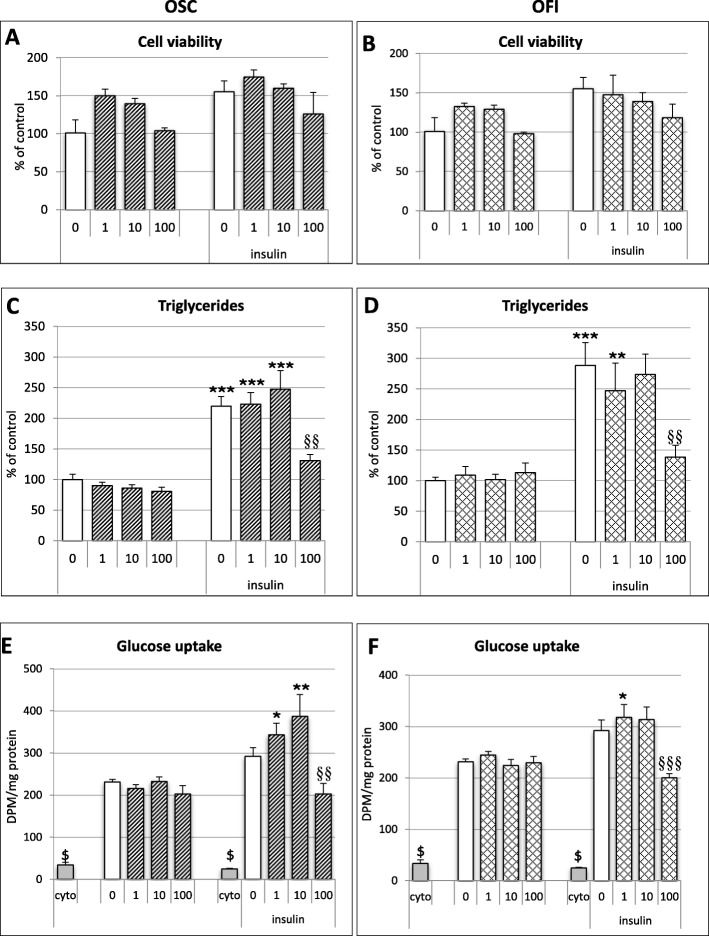
Effects of OCP on cell viability, triglyceride content and glucose uptake in 3 T3-F442A differentiating adipocytes. 3 T3-F442A pre-adipocytes were grown for 10 days in culture medium renewed every 2–3 days. *Opuntia* cladode powders (OCP), *O. streptacantha* (OSC) and *O. ficus-indica* (OFI) were diluted directly in the culture medium (concentrations used were 1, 10, 100 μg/mL). **a**, **b **Cell viability was assessed in pre-adipocytes (left panel) and differentiated adipocytes (induced by 50 nM insulin; right panel) using the MTT assay after treatment with OSC (**a**); OFI (**b**). Graphs show the average values of three independent experiments. Results are expressed as mean percentage of the control (cells without OCP) in non-differentiated adipocytes. Statistical analyses involved ANOVA, followed by Newman–Keuls post hoc test. (**c**, **d**) TG content of 3 T3-F442A adipocytes was evaluated in pre-adipocytes (left panels) and differentiated adipocytes (right panels) treated with OSC (**c**); OFI(**d**). Data represent the mean percentage levels of the control (without OCP) in non-differentiated adipocytes normalised to protein content. Statistical analyses involved ANOVA, followed by Newman–Keuls post hoc test. **p < 0.01, ***p < 0.005 indicates significant difference from control without insulin and treatment; §§ *p* < 0.01 indicates significant difference between groups in insulin-treated cells. (**e**, **f**) Insulin-stimulated uptake of glucose in 3 T3-F442A adipocytes and effect of OCP. Glucose uptake was evaluated in pre-adipocytes (left panels) and differentiated adipocytes (right panels), with/without OCP treatment with OSC (**c**);OFI (**d**). Data are the mean percentage levels of the control (without OCP) in non-differentiated adipocytes normalised to protein content. Cytochalasin (cyto) 10 μM, was used as the negative control for glucose uptake. Statistical analyses involved ANOVA, followed by Newman–Keuls post hoc test. ^$^*p* < 0.005 significantly different from all groups; **p < 0.01, *p < 0.05 significant difference from control without insulin and treatment; ^§§^*p* < 0.01, ^§§§^*p* < 0.005 indicates significant difference between groups in insulin-treated cells

**Fig. 2:**
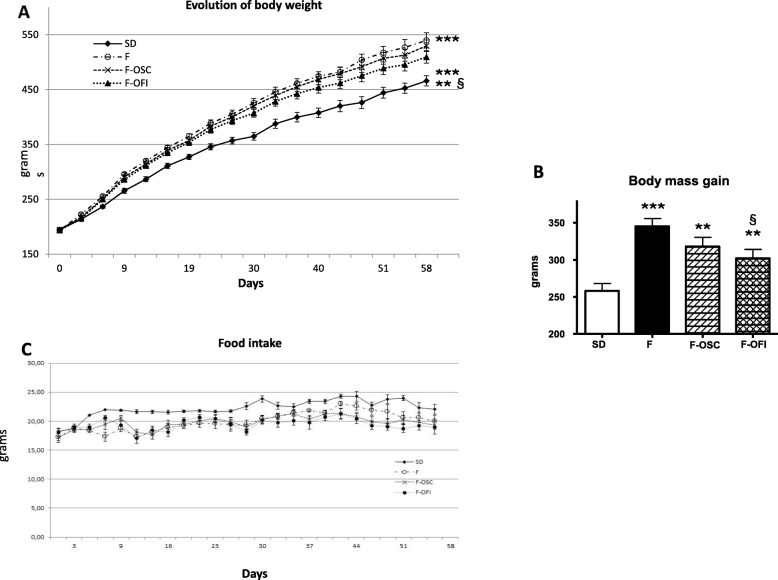
Effects of OCP on body mass. The effects of *Opuntia* cladode powders (OCP) were evaluated in HFD-fed rats supplemented with 0.5% *O. streptacantha* and *O. ficus-indica*, respectively (F-OSC, F-OFI), compared with OCP-untreated controls (F). Group SD received a standard diet. **a** Body mass evolution was recorded weekly over 8 weeks. An average of the body weight of each group is expressed by mean ± SEM. **b** The body mass gain was calculated for each animal as the difference of its body mass between the start (day 0) and end (day 60) of the experiment. Data represent mean ± SEM from each group. **c** The food intake was recorded for each animal over 8 weeks. All data represent mean ± SEM. Each group was composed of 10 rats. Statistical analyses involved ANOVA, followed by Newman–Keuls post hoc test. **p < 0.01, ***p < 0.005 indicates significant difference from group SD; ^§^*p* < 0.05 denotes significant difference from group F

All data were expressed as mean ± standard error of the mean (SEM) of three (or more) independent experiments (cell experiments) or 10 rats per group (in vivo experiments). Statistical significance was determined by one-way analysis of variance (ANOVA), followed by Newman–Keulspost hoc test, using GraphPad Prism software. Statistical significance was indicated by *p < 0.05, **p < 0.01 and ***p < 0.005. In Fig. 1, ^§§^*p* < 0.01, ^§§§^*p* < 0.005 indicates significant difference between groups in insulin-treated cells. In Figs. 2 and 4 ^§^*p* < 0.05; ^§§^*p* < 0.01, denotes significant difference of F-OCP and F-OFI from the F group.

## Results

### Characterisation of biological compounds in the two *Opuntia* cladode powders (OCP)

For each species (OSC, OFI), young cladodes at a similar maturity stage were collected. The contents of macromolecules, phenolic acids and flavonoids, and the antioxidant capacities of the OCP were analysed in a previous study [12]. The results are summarized in Table 3. The proximal composition showed no differences in protein (11.0 and 11.7%) and fat content (0.62 and 0.68%), but OSC had higher fibre content (6.52%), while OFI presented the highest ash content (14.2%). OSC contained the highest phenolic compound (65.1 μg gallic acid equivalent/g sample) concentration and antioxidant capacity, but no difference in flavonoid content between the two species was observed.

**Table 3:** Proximal composition, phenolic compounds, and antioxidant capacity of both *Opuntia streptacantha* and *Opuntia ficus-indica* cladode powders

Biomolecules	*Opuntia*		
	*streptacantha*	*ficus-indica*	
Proximal composition (%) ^a^			
Protein	11.0	11.7	
Fat	0.62	0.68	
Fibre	6.52	5.63	$
Ash	12.6	14.2	$
Phenolic acids^b^	65.1	56.7	$
Flavonoids^c^	19.0	20.4	
Antioxidant capacity^d^	897.8	659.4	$

^a^values are the mean of triplicates on dry weight basis.

^b^as μg of gallic acid/g sample;

^c^as μg of quercetin/g sample;

^d^as μmol of Trolox/g sample.

$ statistically differences between means.

### Effects of *Opuntia* cladode powders (OCP) on 3 T3-F442A pre-adipocyte differentiation

#### Cytotoxic effects of Opuntia cladode powders (OCP)

To evaluate the potential cytotoxic effects of OCP, pre-adipocytes were differentiated into mature adipocytes for 10 days with 50 nM insulin, in the presence of various concentrations of OCP (0, 1, 10 and 100 μM). As shown in Fig. 1, treatment with OSC (Fig. 1a) and OFI (Fig. 1b) had no markedly effect on cell viability, around 100% of the control, even with the highest OSC concentration (100 μM). At lower concentrations (1 and 10 μM), the cell viability was increased, possibly due to a mitogenic effect of OCP.

#### Opuntia cladode powders (OCP) inhibit triglyceride (TG) storage in adipocytes

To examine the effects of OCP on the differentiation of 3 T3-F442A pre-adipocytes in adipocytes, confluent cells were treated with increasing concentrations of OCP. As shown in Fig. 1c, d, the TG content was not affected by OSC and OFI, in undifferentiated cells (without insulin). Differentiated cells exhibited a significant increase in intracellular TG content (about 250%) which was significantly reduced in cells treated with 100 μM OCP (OSC, 60%; OFI, 50%; *p* < 0.01). No effect was observed for lower OCP concentrations (1 and 10 μM).

#### Effects of Opuntia cladode powders (OCP) on cellular uptake of glucose

The differentiation of pre-adipocytes into mature adipocytes is characterized by a strong increase in glucose uptake in response to insulin [26]. As high OCP concentrations tend to decrease the differentiation of 3 T3-F442A cells into mature adipocytes, we checked the effect of OCP on the glucose uptake evoked by insulin in undifferentiated and differentiated cells. The results presented in Fig. 1 e,f showed that insulin stimulated the uptake of glucose under our experimental conditions. *Opuntia* powders had no noticeable effect on glucose uptake in the absence of insulin. When cells were differentiated by insulin, low-to-moderate concentrations of OCP slightly increased the uptake of glucose elicited by insulin. However, when cells were incubated with high OSC and OFI concentrations (100 μg/mL) during the differentiation process, the glucose uptake remained similar to that observed in undifferentiated 3 T3-F442A cells (70% OSC and 60% OFI of the control cells treated with insulin but without OCP; *p* < 0.001). Altogether, these results (low TG levels and low glucose uptake) suggested that OCP prevented or reduced the differentiation of 3 T3-F442A cells into mature adipocytes.

### *Opuntia* cladode powders (OCP) supplementation in diet prevented HFD-induced obesity

#### Body weight and food intake

**Fig. 3:**
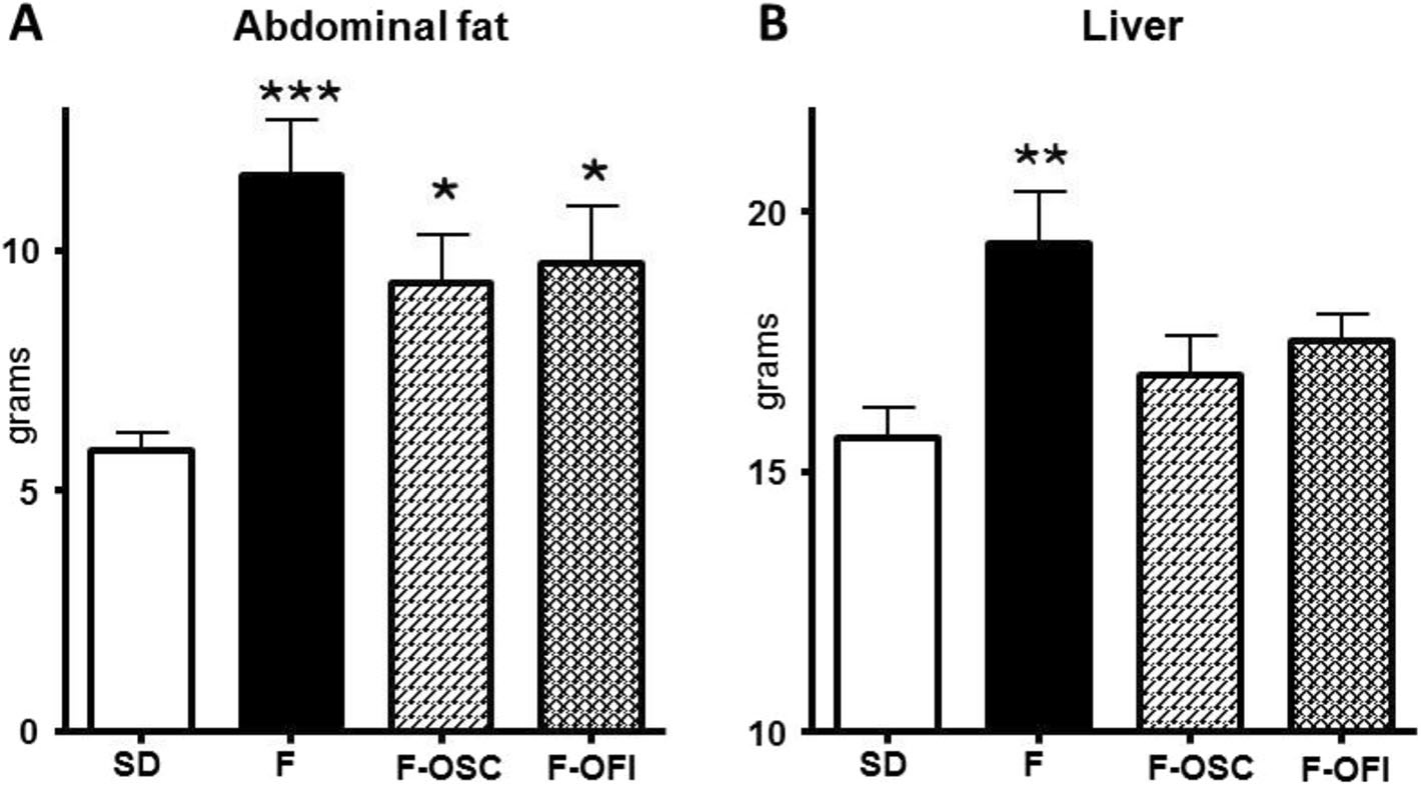
Effects of *Opuntia* cladode powders (OCP) supplementation on relative abdominal fat mass (**a**) and liver mass (**b**). Data represent mean ± SEM. Statistical analysis involved ANOVA, followed by Newman–Keuls post hoc test. *p < 0.05, **p < 0.01, ***p < 0.005 indicates significant difference from the group fed the standard diet (SD)

To investigate whether OCP may modulate obesity in an animal model, Sprague–Dawley rats were HFD-fed supplemented with 0.5% OCP. The percentage of powders used in rat diets was chosen as an average of the percentage used in previous studies of 0.25 to 1% [14, 15, 22, 27]. OSC and OFI were given for 8 weeks and compared with HFD given without supplement and standard diet (SD). Growth parameters were evaluated throughout the 60 days of OCP treatment for each rat. Initial body weights were not dramatically different among the groups. After 8 weeks, the final body weights were significantly higher in the three HFD groups (F, F-OSC, F-OFI), when compared to the control (SD) group (Fig. 2a; *p* < 0.005). HFD-fed rats supplemented with OFI (F-OFI) exhibited a body mass significantly lower (87.5%; *p* < 0.05) when compared to group F (Fig. 2a). Likewise, the final body mass was lower in group F-OSC*,* but not significant. The body mass gain (Fig. 2b), representing the average of individual differences of body mass from the initial and final body mass, presented a significant difference between the HFD diet and standard diet SD (F; *p* < 0.005, OCP; *p* < 0.01). A significantly lower gain in body mass was observed for OFI-supplemented rats when compared to the control group (F) (*p* < 0.05). No significant differences in food intake were observed between the three HFD groups throughout the experiment, indicating that the reduction in body weight gain in the OFI group was not due to reduced food intake (Fig. 2c). It is to note that the weekly food intake was higher in the SD group than for the HFD (F) groups (Fig. 2c). The weight of abdominal fat was significantly higher in HFD than SD groups (200%; *p* < 0.005). Both OCP reduced this increment by about 80% between F-OSC and F-OFI vs SD; *p* < 0.05; (Fig. 3a). Same results were observed for liver weight (Fig. 3b), even if the slight decreases observed in groups F-OSC and F-OFI were not markedly different from group F and the SD group.

#### Metabolic parameters

**Fig. 4:**
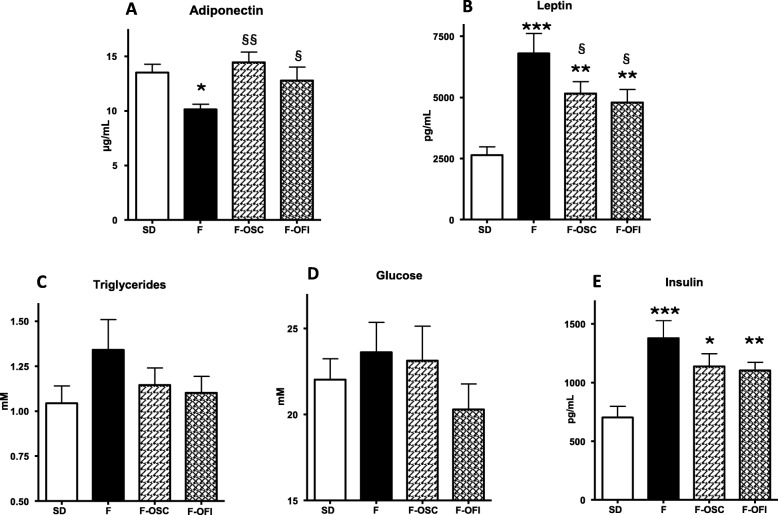
Effect of OCP supplementation on various biological parameters in rat serum. Rats were fed with (F) or without (SD) a high-fat diet, supplemented with *Opuntia* cladode powders (OCP) from *O. streptacantha* and *O. ficus-indica*, respectively (F-OSC, F-OFI). **a** Adiponectin (μg/mL); (**b**) leptin (pg/mL); (**c**) triglycerides (mM); (**d**) glucose (mM); (**e**) insulin (pg/mL). All data represent mean ± SEM. Each group was composed of 10 rats. Statistical analyses involved ANOVA, followed by Newman–Keuls post hoc test. *p < 0.05, **p < 0.01, ***p < 0.005 indicates significant difference from group SD;^§^*p* < 0.05, ^§§^*p* < 0.01 significant difference from group F

The effects of OCP supplementation were investigated on metabolic parameters, by measuring the serum levels of obesity-related markers (Fig. 4). As shown in Fig. 4 a, b, the increase in adiposity was associated with a decrease in adiponectin level in group F (F = 75% vs SD; *p* < 0.01) and an increase in leptin level (F = 260% vs SD; *p* < 0.005). OCP supplementation in food restored the adiponectin level (F-OSC = 143%; p < 0.01, F-OFI = 126%; p < 0.05, compared to the F group). A significant increase in leptin level was observed in the 3 HFD groups (when compared to SD, F-OSC = 195%; *p* < 0.01 and F-OFI = 180%; *p* < 0.01, as compared with 260% in F; *p* < 0.005), with a decrease of 25 and 30% for F-OSC and F-OFI respectively compared to the F- group, *p* < 0.05).

HFD diet slightly increased the levels of circulating TG (130%), and this was reversed by both OCP supplementation (Fig. 4c) though not significantly. Same observations could be done for circulating glucose levels. OCP supplementation tended to decrease TG and glucose levels (Fig. 4c,d). Likewise, circulating insulin levels (Fig. 4e) were increased in group F (195%; *p* < 0.005) by comparison with the SD group, and were reduced by OCP supplementation (18 and 20% for F-OSC and F-OFI respectively).

### Effects of OCP supplementation on liver and inflammation

**Fig. 5:**
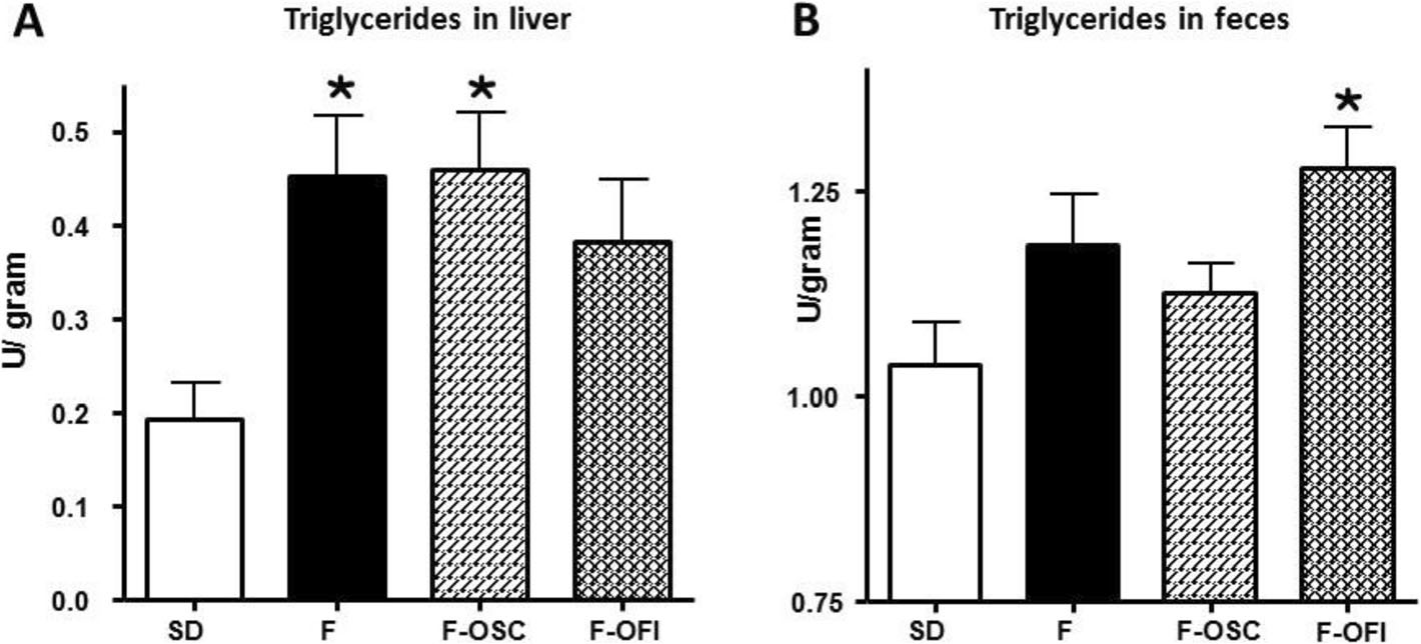
Triglyceride content in liver (**a**) and faeces (**b**). Rats were fed with (F) or without (SD) a high-fat diet, supplemented with *Opuntia* cladode powders (OCP) from *O. streptacantha*and *O. ficus-indica*, respectively (F-OSC, F-OFI). Triglyceride concentrations were reported relative to liver or faeces weight. Data represent mean ± SEM. Statistical analysis involved ANOVA, followed by Newman–Keuls post hoc test. *p < 0.05 indicates significant difference from group SD

HFD causes hepatic inflammation and steatosis, which can further lead to non-alcoholic steatohepatitis and non-alcoholic fatty liver disease (NAFLD) [28]. To evaluate the potential toxicity of HFD and OCP supplementation, serum markers for liver injury and liver TG content were measured in our rat model. No noticeable differences were observed among the different groups concerning the levels of AST and ALT and inflammation markers, such as MCP-1 (Table 4) and CRP (data not shown), Therefore, neither HFD nor OCP supplementations caused noticeable adverse toxic effects in rats. The hepatic TG content (Fig. 5a) was significantly increased due to HFD diet (HFD groups, 235% vs SD; *p* < 0.05). OFI supplementation slightly reduced the TG content (85% of group F; *p* < 0.05), but not significantly.

**Table 4:** Serum hepatic markers

Marker	SD	F	F-OSC	F-OFI
AST (U/L)	32.6 ± 2.42	30.3 ± 1.78	35.0 ± 3.26	37.0 ± 4.61
ALT(U/L)	78.3 ± 3.91	64.6 ± 1.99	78.2 ± 6.45	72.8 ± 4.49
MCP-1 (pg/mL)	817 ± 49.2	881 ± 37.9	803 ± 53.8	877 ± 31.5

SD = standard diet; F = high fat diet; F-OSC =*Opuntia streptacantha* cladode powder; F-OFI = *Opuntia ficus-indica* cladode powder. AST = aspartate aminotransferase. ALT = alanine aminotransferase. MCP-1 = monocyte chemoattractant protein 1. Results are mean of 10 animals ± SEM. *No significant difference were observed*.

### Triglyceride (TG) evaluation in faeces

Our results indicated that HFD-fed rats supplemented with OCP, presented a lower body mass and reduced TG levels in the serum and the liver when compared to HFD (F) control rats. To understand the mechanisms implicated in the decreased TG levels, the TG content of rat faeces was measured 3 days before the end of experiments. The results in Fig. 5b, indicate that TG concentration was increased in faeces from the three HFD groups, particularly in the group F- OFI (123%; *p* < 0.05), when compared to the SD group. However the TG content in faeces from this group was comparable to the F and F-OSC groups.

## Discussion

In the present study, we investigated the effects on obesity of two OCP, the wildest OSC and the most domesticated OFI, using 3 T3-F442A adipocytes cells and HFD obese rats.

Adipocytes play a central role in the maintenance of lipid homeostasis and energy balance by storing TG or releasing free fatty acids in response to changes in energy demand. These cells represent a good model for investigating molecules able to reduce obesity via an impairment of differentiation and adipogenesis. In this work, we used the well-characterised murine pre-adipose 3 T3-F442A cell line for exploring the effects of OCP on adipogenesis [29, 30]. Our results indicate that cladode powders from two different species effectively alter adipogenesis by reducing TG accumulation during the differentiation process, without generating cytotoxicity. This observation is in agreement with previous studies showing that OCP (powders or ethanol extracts) may reduce adipocyte differentiation and adipogenesis [22, 27, 31, 32]. The differentiation of 3 T3-F442A pre-adipocytes in mature adipocytes by insulin includes an increase in both TG content and glucose uptake [33]. Our data show that high OCP concentrations inhibited the uptake of glucose elicited by insulin in differentiating cells. It is important to emphasize that this low glucose uptake was not associated with an increased TG storage, which would be indicative of insulin resistance. In contrast, the association of low TG content and low glucose uptake elicited by OCP in conditions of preadipocyte differentiation by insulin, supports an inhibitory effect of these agents on the differentiating process into mature adipocytes. Accordingly, it could be hypothesised that OCP and especially OFI treatment, may reduce adipocyte storage of TG and consequently adipocyte hypertrophy. Our data show that both OCP (with OFI being the most effective) exert anti-adipogenic effects in the 3 T3-F442A cell line, at concentrations effective against low-density lipoprotein oxidation, foam cell formation, and atherogenesis in apoE-knockout mice, and in cellular models for colon cancer studies in vitro [13, 14]. OCP effects could be compared with those of resveratrol, which exerts anti-obesity effects by inhibiting glucose utilisation in 3 T3-F442A cell line [34].

The chemical composition and the presence of phenolic compounds in the different *Opuntia* species has been previously reported [12]. Among the molecules identified in OCP, flavonoids, quercetin, kaempferols and isorhamnetin, could be implicated in weight loss [15, 16, 22, 35, 36]. Our previous studies indicated that the levels of flavonoids, quercetin, kaempferols and isorhamnetin detected by mass spectroscopy (LC-MS/MS), are higher in OFI than in OSC [12], which may explain the higher efficacy of this OCP in reducing HFD-induced weight gain. It is to note that low OCP concentrations were not active, possibly due to very low concentrations of the different phenolic compounds in the powders. These data are in agreement with studies reported by Lee et al. [16, 36], who showed that purified molecules (isorhamnetin or kampferol) may inhibit adipocyte differentiation and lipid accumulation. Kampferol blocked the phosphorylation of AKT and mTOR, acting on early adipogenic factors, which resulted in attenuation of late adipogenic factors such as C/EBP-α and PPARγ. The same genes (C/EBP-α and PPARγ) and their target genes (LPL, aP2, LXR) were identified as isorhamnetin targets. Thus, these active compounds being present in O*puntia* cladodes, they could act at the molecular level by regulating lipid metabolism. As OFI is the most domesticated cultivar, it could be of interest to select OFI species with higher phenolic compound content to improve their anti-obesity properties.

In our study, HFD-fed rats gained markedly more weight than those fed a normal diet, confirming that diet-induced obesity was successful. No noticeable difference was observed in food intake among all groups. Interestingly, OCP supplementation (F-OSC, F-OFI) prevented the weight of gain of animals with comparable food intake. Furthermore, OCP tended to reduce abdominal fat development over the 8 weeks of the diet without reducing food intake. Thus, we can conclude that OCP supplement in food could decrease the body weight gain by repressing the expansion of adipose tissue mass.

Leptin is a secreted peptide encoded by the *obese* gene and produced primarily by adipose cells. It plays a vital role in controlling body weight, presumably by acting in the hypothalamus to suppress appetite. Body fat is the most important determinant of circulating leptin levels, but other factors also acutely regulate the production and secretion of leptin, for instance, fasting decreases leptin, while refeeding restores the circulating leptin in both mice and humans [37]. Adiponectin is also secreted from adipocytes, and low circulating levels have been epidemiologically associated with obesity, insulin resistance, type 2 diabetes and cardiovascular diseases. Adiponectin promotes cell proliferation and differentiation of pre-adipocytes into adipocytes, augmenting programmed gene expression responsible for adipogenesis, and increasing lipid content and insulin responsiveness of the glucose transport system in adipocytes [38]. Circulating leptin levels are increased in HFD-fed animals, in parallel with a decrease in circulating adiponectin concentrations. In our study, serum adiponectin concentration was noticeably reduced in HFD-fed rats, which was reversed by OCP supplementation, suggesting that OCP treatment activated the adipocyte production of adiponectin. Moreover, serum leptin levels were increased in all HFD groups compared with the standard diet (SD), but the leptin levels were lower in the F-OSC and F-OFI groups. Leptin is known to regulate food intake and stimulates energy expenditure. As no differences were observed in food intake, the anti-obesity effects of OCP could be related to increased leptin sensitivity and modifications in energy expenditure. All these results are in agreement with an improvement in blood parameters related to obesity due to OCP supplementation in HFD. The same results were observed in a mouse model of diet-induced obesity, using isorhamnetin glycosides extracted from OFI [15], or using different bioactive compounds extracted from seaweed or ginseng leaf or Korean red ginseng on insulin sensitivity [8, 33].

A HFD is known to induce NAFLD in animal models and humans by causing fat deposition in the liver [39, 40]. NAFLD is closely associated with obesity. In our HFD-fed rat model of obesity, we showed that OCP supplement in the diet tends to lower the liver weight, which can be correlated to less TG storage in the liver. Similarly, Moran-Ramos et al. demonstrated that *Opuntia* cladode consumption attenuates hepatic steatosis in obese Zucker rats [18], and other studies based on HFD supplemented with quercetin [35] or *Vignanakashimae* extracts (another flavonoid-rich plant) [32] also resulted in a lowering of body weight gain and hepatic lipid accumulation. Taken together, these results suggest that OCP could be efficient against fatty liver in HFD obese rats. It is noteworthy that OCP treatment did not cause any detectable adverse toxic effects on the liver.

Uebelhack et al. [9] and Chong et al. [41] illustrated that the effects of *Opuntia*-derived fibers act in reducing dietary fat absorption in human volunteers, by binding to dietary fat and increasing its excretion in faeces, probably by decreasing fat intestinal absorption. To further understand the mechanisms explaining the lower weight gain induced by *Opuntia* supplementation in a HFD, we evaluated the faecal excretion of fats in our rat model, which was markedly increased in rats fed a HFD, and was much more pronounced when rats were supplemented with OFI. These findings support the hypothesis that the effects of OCP on weight are achieved by reducing dietary fat absorption, leading to lower energy intake and thus, to a lesser weight gain. Finally, nopal anti-inflammatory effects had been identified. Bouhini et al showed a reduction in low-grade chronic inflammation associated with obesity, this could be due to the effect of nopal fibers on the gut microbiota [17, 20].

## Conclusions

Our study was performed using a rat model for obesity and a cellular model, which permits to reduce the number of animals used and obtain information about the cellular mode of action. However, using animals highlighted the decreased intestinal fat absorption and the non-toxic effects of dietary *Opuntia* powders, suggesting their potential to be used in the human diet. The mode of action of *Opuntia* in obesity management need to be further analyzed but it appears that some effects could be observed at the molecular level by regulating the adipocyte differentiation genes pathway, by reducing fat absorption as well as a possible modification of the gut microbiota. In conclusion, our data suggest that OFI cladode consumption may be useful in the management of obesity and the prevention of hepatic diseases (NAFLD) related to obesity.

## Data Availability

The datasets used and/or analysed during the current study are available from the corresponding author on reasonable request.

## Abbreviations

HFD: High-fat diet
NAFLD: Non-alcoholic fatty liver disease
OCP: *Opuntia* cladode powders
OFI: *Opuntia ficus-indica*
OSC: *Opuntia streptacantha*
TG: Triglycerides
